# The 35th annual advances in contrast ultrasound international bubble conference, Chicago 2021: synopsis and take-home messages

**DOI:** 10.1186/s44156-022-00002-9

**Published:** 2022-06-06

**Authors:** Michael Dunleavy, Alan Goldberg, Steven Feinstein, Stephanie Wilson, Sharon Mulvagh, Petros Nihoyannopoulos

**Affiliations:** 1 Rush University Medical Center, Internal Medicine, Chicago, IL USA; 2 Alberta Health Services, Radiology and Medicine, Edmonton, Canada; 3 Mayo Clinic, Cardiovascular Medicine, Rochester, MN USA; 4 Imperial College London, NHLI, National Heart and Lung Institute, London, UK

**Keywords:** Contrast Echocardiography, Image Quality, Preclinical, Ultrasonography

## Abstract

The 35th Annual Advances in Contrast Ultrasound International Bubble Conference convened in Chicago, IL, USA, on September 30th to October 1st, 2021. It featured a range of novel research from animal studies to clinical applications in multiple organ systems, demonstrating the utility of contrast enhanced ultrasound (CEUS). A multidisciplinary group of experts on the use of CEUS, including physicians, basic scientists, engineers, and industry partners, convened to discuss cutting edge research and new applications for CEUS. The conference demonstrated the wide range of CEUS uses and potential uses, including cardiac risk stratification, sonothrombolysis, peripheral vascular reperfusion, liver and renal mass evaluation, lymphatic evaluation, sentinel node identification, and CEUS use in pediatrics. The International Contrast Ultrasound Society uses this information to continue advocating for the safe and appropriate use of CEUS.

## Improvements in echocardiography images and Major Adverse Cardiovascular Events (MACE) risk stratification 

Ultrasound enhancing agents are effective at decreasing uninterpretable echocardiogram studies. Dr. Jordan Storm of Harvard Medical School presented a new algorithm to increase ultrasound enhancing agent use for prospective testing. Patients with a high probability of needing CEUS (> 50%), based on height, heart rate, and age, have an IV placed before the scan. The sonographer can then determine at the point of care if CEUS is needed, improving its utilization.

CEUS can also be used to improve stress echocardiogram assessment for MACE. While stress echocardiography has good sensitivity for coronary stenosis, it has poor performance in predicting cardiovascular outcomes. Dr. Amer Johri from Queens University in Kingston, Canada discussed CIRCE, a multicenter clinical trial assessing the use of inter-plaque neovascularization (IPN) to improve cardiac risk prediction. In patients with a negative stress echocardiogram, the patient is deemed “low risk” if there is no plaque, “intermediate risk” if there is plaque but no IPN, and “high risk” if there is plaque with IPN. The current 3-year MACE sensitivity for a stress echocardiogram alone is approximately 60%, but the investigators hope a carotid ultrasound without plaque will improve that to 80%.

## Sonothrombolysis and peripheral vascular reperfusion

Dr. Harald Becher of the Alberta Heart Institute discussed use of high mechanical index ultrasound waves for thrombolysis in ST-elevation MI (STEMI). In a pilot study of 15 patients who underwent sonothrombolysis, there was Thrombolysis In Myocardial Infarction (TIMI)-3 flow in all patients and 93.3% had ST resolution post-percutaneous coronary intervention (PCI). The ejection fraction (EF) was at least 50% in 4/5ths of patients, with only one patient having an EF less than 40%. Dr. Becher also discussed the SONOSTEMI-Lysis trial, in which patients are administered thrombolytic therapy, then are randomized to receive sonothrombolysis or not. At 90 min post thrombolysis, the decision is made for urgent PCI or delayed PCI based on follow up ECG. The goal is to improve clinical outcomes, as well as decrease the rate of rescue PCI and necessary PCI during nightshift and weekends.

Dr. Tom Porter from the University of Nebraska discussed a multicenter post-PCI sonothrombolysis trial for anterior wall STEMI, comparing very low mechanical index imaging with intermittent High Mechanical Impulse Index (HMII). In this pilot study, there was a trend towards significance in microvascular obstruction improvement and a statistically significant improvement in the LVEF in the HMII group after 6 weeks.

Phase shift microbubbles (PSMBs) can also be used for sonoreperfusion therapy for microvascular obstruction. Microbubble-mediated sonothrombolysis dissolves the clot and restores blood flow by penetrating into the structure of the clot, leading to mechanical lysis. Acoustically responsive PSMBs, which are 1/10th the size of a normal microbubble, can be molecularly targeted to fibrin for use in sonothrombolysis. Dr. Evan Unger from the University of Arizona showed fibrin targeted PSMBs performed extremely well for sonothrombolysis in a rodent model. The use of catheter-based ultrasound for microbubbles and PSMBs provided excellent flow restoration at 75 min while reducing the thrombolytic dose in a porcine deep vein thrombosis model, further demonstrating the potential of PSMBs.

RBC cavitation has been shown to augment flow in peripheral artery disease. For years the underlying hypothesis was that bubble vibration led to release of nitrous oxide (NO) and adenosine triphosphate (ATP) from endothelial cells, thus increasing blood flow. However, Dr. Jonathan Linder from the University of Oregon showed red blood cells release ATP and NO when microbubbles near them are vibrated at high mechanical index. In both mouse and primate models, these vasodilator components travel downstream, augmenting flow outside of the ultrasound beam. Additionally, he showed the same phenomenon occurs in catheters delivering ultrasound in a pulmonary embolism model.

**Fig. 1:**
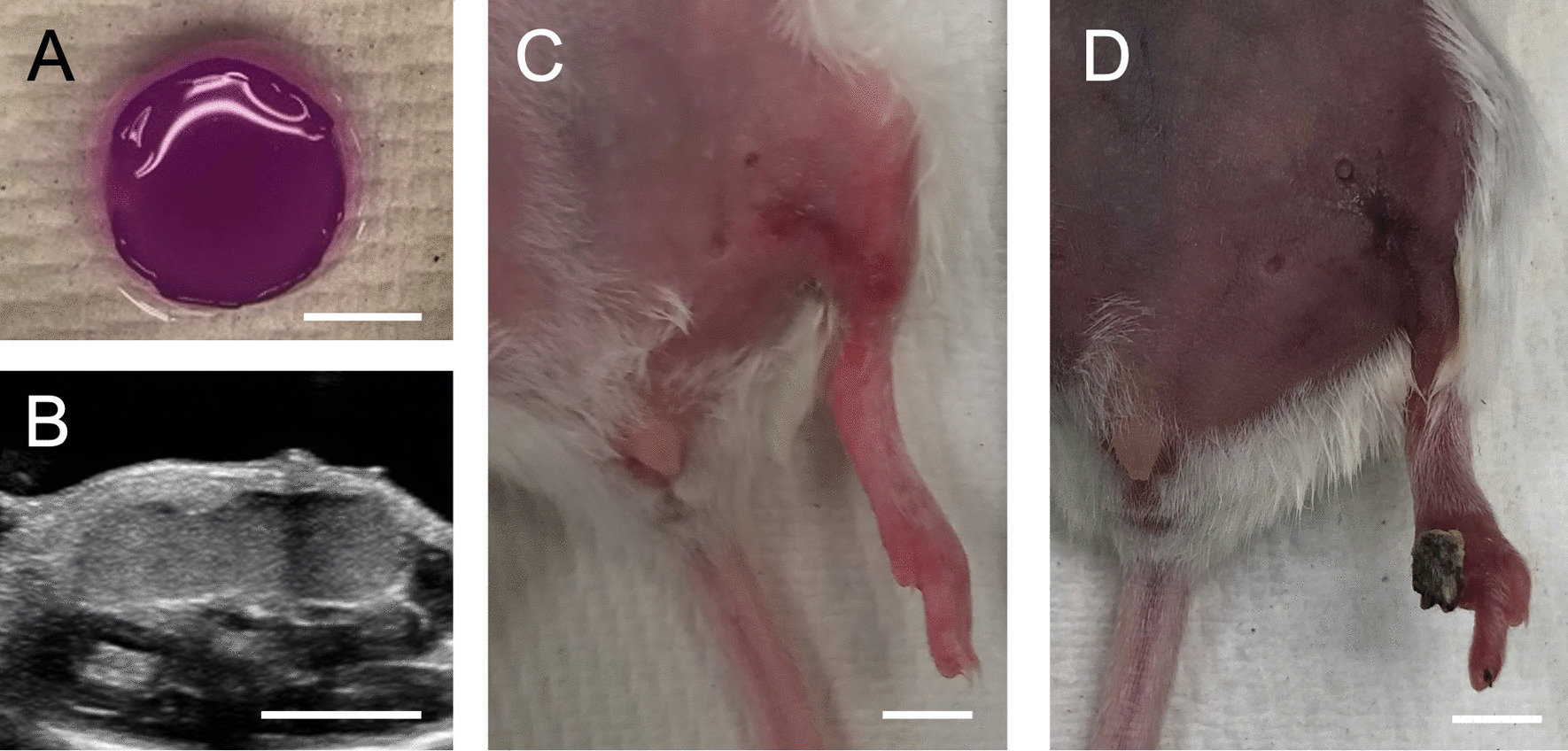
Optical image (**A**) and in situ, B-mode image (**B**) of an acoustically responsive scaffold. Mice receiving an implant in conjunction with ultrasound (**C**) exhibited less necrosis than mice receiving only an implant (**D**). Scale bar in all panels: 5 mm

Approximately one-fourth of critical limb ischemia is not eligible for surgical or endovascular therapy. However, microbubble precursors can deliver bioactive molecules for therapeutic angiogenesis. Acoustically responsive scaffolds are microbubble precursors with growth factor encapsulated in fibrin. Dr. Mario Fabiilli from the University of Michigan showed this scaffold can be modulated using focused ultrasound, releasing the growth factor. In a mouse model, the femoral artery is ligated then implanted with an acoustically responsive scaffold. The mice with the implant plus ultrasound had the best recovery and much higher blood vessel density post-surgery, suggesting their potential future role in critical limb ischemia (Fig. 1). This data has since been published in the Journal of Controlled Release [1].

## Liver mass evaluation

Dr. Stephanie Wilson of the University of Calgary discussed the benefits of contrast enhanced ultrasound for liver mass evaluation. In particular, CEUS is useful in patients who are unable to receive MRI or CT with contrast, since those modalities often show solid organ pathology through intravenous contrast. Since most incidental lesions are benign, a very low risk, cost-effective option such as contrast enhanced ultrasound is an excellent choice for liver mass evaluation.

**Fig. 2:**
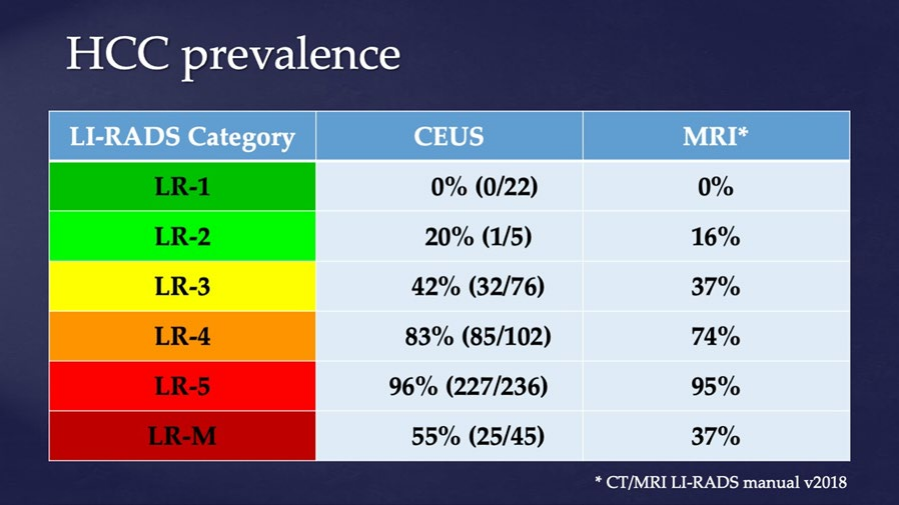
HCC prevalence by LI-RADS category using CEUS compared to tissue histology and imaging follow-up as reference standard. CT/MRI data from CT/MRI LI-RADS manual v2018

The Liver Reporting and Data System (LI-RADS) was developed to be highly specific for HCC, utilizing arterial phase hyper-enhancement, washout, and tumor size for differentiation. Dr. Andrej Lyshchick of Thomas Jefferson University presented a prospective study validating the use of CEUS in LI-RADS and determining the diagnostic performance of CEUS for HCC detection in patients at risk for HCC with untreated focal liver lesions. CEUS had similar results to MRI and CT in all categories including LR-5, which is highly specific for HCC and is considered diagnostic. The specificity for CEUS to determine LR-5 masses was 96.2%, confirming the high clinical value of CEUS for non-invasive HCC diagnosis (Fig. 2).

The LI-RADS score uses a qualitative and semi-qualitative hypothetical time intensity curve to assess washout of contrast from the tumor. Dr. Michael Averkiou of the University of Washington discussed work to improve the early detection of HCC by collecting time intensity curves to redistribute indeterminate masses or those suggestive of HCC, to be either unlikely HCC or LR-5. They used respiratory gating, an articulated arm, and motion compensation, allowing for the detection of mild washout that might be missed in a qualitative evaluation. Thus, they were able to change what was previously a qualitative assessment into a quantitative assessment with CEUS.

CEUS is also useful in surveillance after HCC treatment with ablation or ethanol injection. Recurrence is common, so prompt detection is important. Dr. Stephanie Wilson discussed a new secondary surveillance algorithm after ablative therapy that has been implemented at the University of Calgary. An MRI was performed one month after ablation, then every 3 months thereafter, alternating between CEUS and MRI. Over half of the recurrence detected was found using CEUS. Intrazonal recurrence was detected 10 times using CEUS, compared with only 3 times using MRI. Additionally, there were 17 suspicious masses on MRI in which CEUS provided the diagnosis, compared to only 3 tumors seen with CEUS in which MRI was needed to accurately differentiate.

**Fig. 3:**
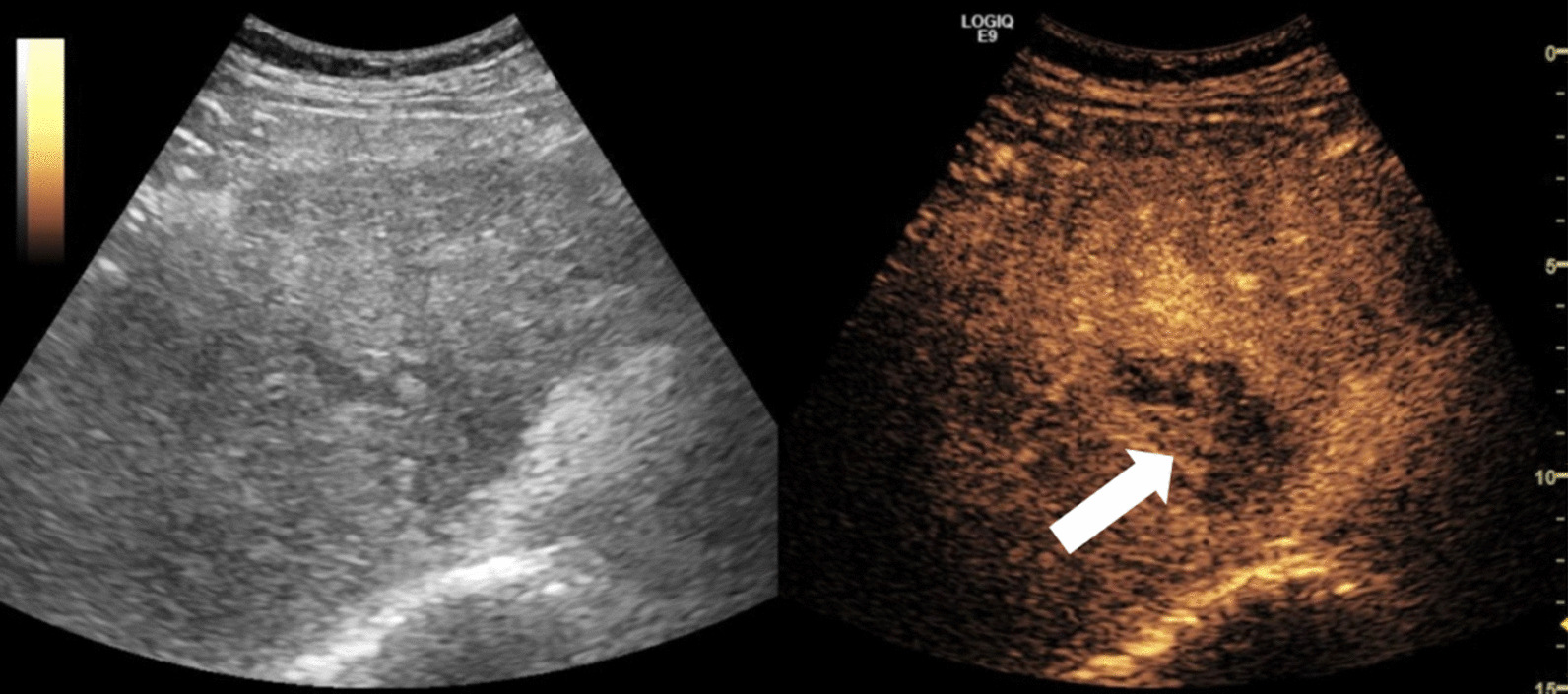
Example CEUS image from a male HCV patient with 4 cm HCC in segment 8 two weeks post transarterial chemoembolization. Nodular peripheral enhancement is observed on CEUS (white arrow), indicating residual viable tumor. Clinical contrast enhanced MRI was obtained 5 weeks post treatment and read as equivocal. Viable tumor was later confirmed via angiography during retreatment

Transarterial Chemoembolization (TACE) is a local treatment used in patients with HCC to downstage disease and as a bridge to transplant. Dr. John Eisenbrey from Thomas Jefferson University presented a study on the use of CEUS in monitoring need for retreatment after TACE (Fig. 3). CEUS detected residual HCC 3–4 weeks earlier than the current clinical standard of MRI. CEUS was found to have 90–95% sensitivity to detect residual disease, higher sensitivity than MRI.

CEUS also shows promise in patients with Fontan-associated liver disease (FALD). FALD is characterized by arterialized nodules that can appear similar to focal nodular hyperplasia (FNH). Evaluation of these nodules with CT or MRI can be challenging due to the presence of epicardial leads and intra-abdominal devices causing streak artifact. Dr. Yuko Kimo of UC San Diego demonstrated how FNH-like nodules typically demonstrate spoke wheel vessels and centrifugal pattern enhancement, which are well characterized with CEUS due to its high temporo-spatial resolution.

## Kidney mass evaluation

Dr. Stephanie Wilson from the University of Calgary discussed the benefits of CEUS for renal mass evaluation. She showed CEUS has an excellent positive predictive value of 91.5% (161 of 176) and negative predictive value of 100% (420 of 420) for characterizing indeterminate renal masses as benign or malignant. Moreover, Dr. Richard Barr of Northeastern Ohio Medical University showed that approximately two-thirds of indeterminate masses are benign on CEUS, and even one-third of masses considered malignant by Bosniak classification, used to classify renal masses by CT or MRI, are actually benign on CEUS. With more data, a negative CEUS for a renal mass may end the workup, and a positive CEUS could skip biopsy and go straight to surgery.

Dr. Barr then discussed the operational benefits of CEUS to improve classification of cystic renal masses. The only intra-abdominal organ CEUS is approved for is the liver. Off label use is common, but this limits the ability to be well informed in its use. Companies are only able to provide education on FDA approved applications, limiting education on off label use and making initiation of CEUS programs challenging. FDA approval for CEUS whole body imaging would allow for increased utilization of CEUS taking advantage of its many safe applications developed throughout the world.

## Lymphatic evaluation and sentinel node identification

**Fig. 4:**
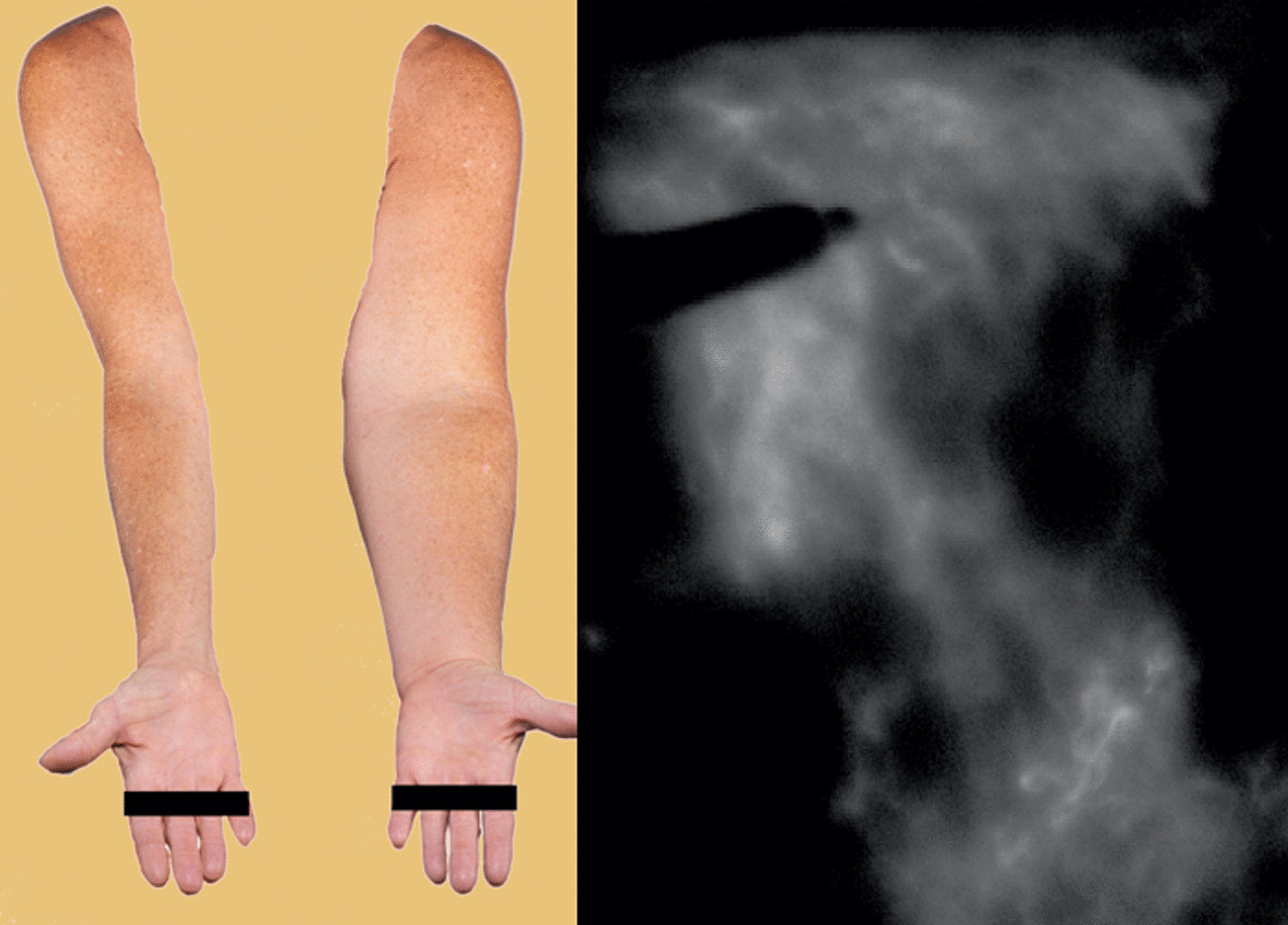
In this patient with left upper extremity lymphedema (left), a marker is used to trace potential lymphatic candidates on the skin after indocyanine green injection demonstrating a background of advanced disease (right)

**Fig. 5:**
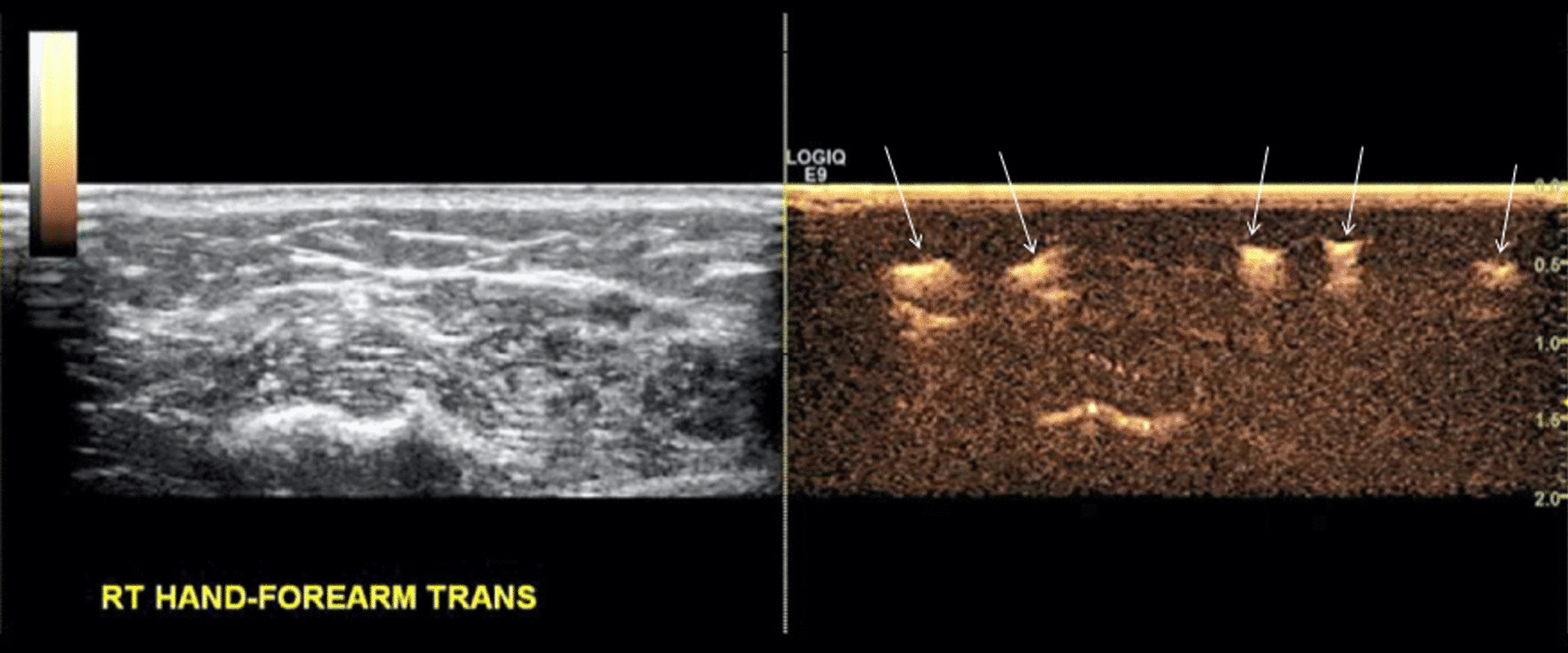
After injection of microbubbles, several lymphatic candidates (right, white arrows) are identified for lymphovenous anastomosis surgery

Dr. Christine Lee of the Mayo Clinic discussed the use of CEUS to assist in surgical procedures to improve lymphatic drainage by anastomosing lymphatic channels to veins. The gold standard for lymphatic mapping, to identify a viable conduit, is indocyanine green. Dr. Lee showed that microbubbles can also identify candidates for surgery (Figs. 4, 5). The surgical success rate is 67% with CEUS, and 50% even when indocyanine green failed to find viable lymphatics. Thus it is plausible microbubbles will become the gold standard moving forward.

Microbubbles can also be used to find sentinel lymph nodes in breast cancer. The current standards of care are blue dye injection or radioactive tracer injection prior to surgery. However, Dr. Flemming Forsberg from Thomas Jefferson University in Philadelphia showed microbubbles injected around the tumor are superior for identifying sentinel lymph nodes. In his study, of the 31 lymph nodes with metastasis on histology, all 31 were detected with CEUS, but only 14 were detected with blue dye and 20 with radiotracer.

## CEUS in pediatrics

**Fig. 6:**
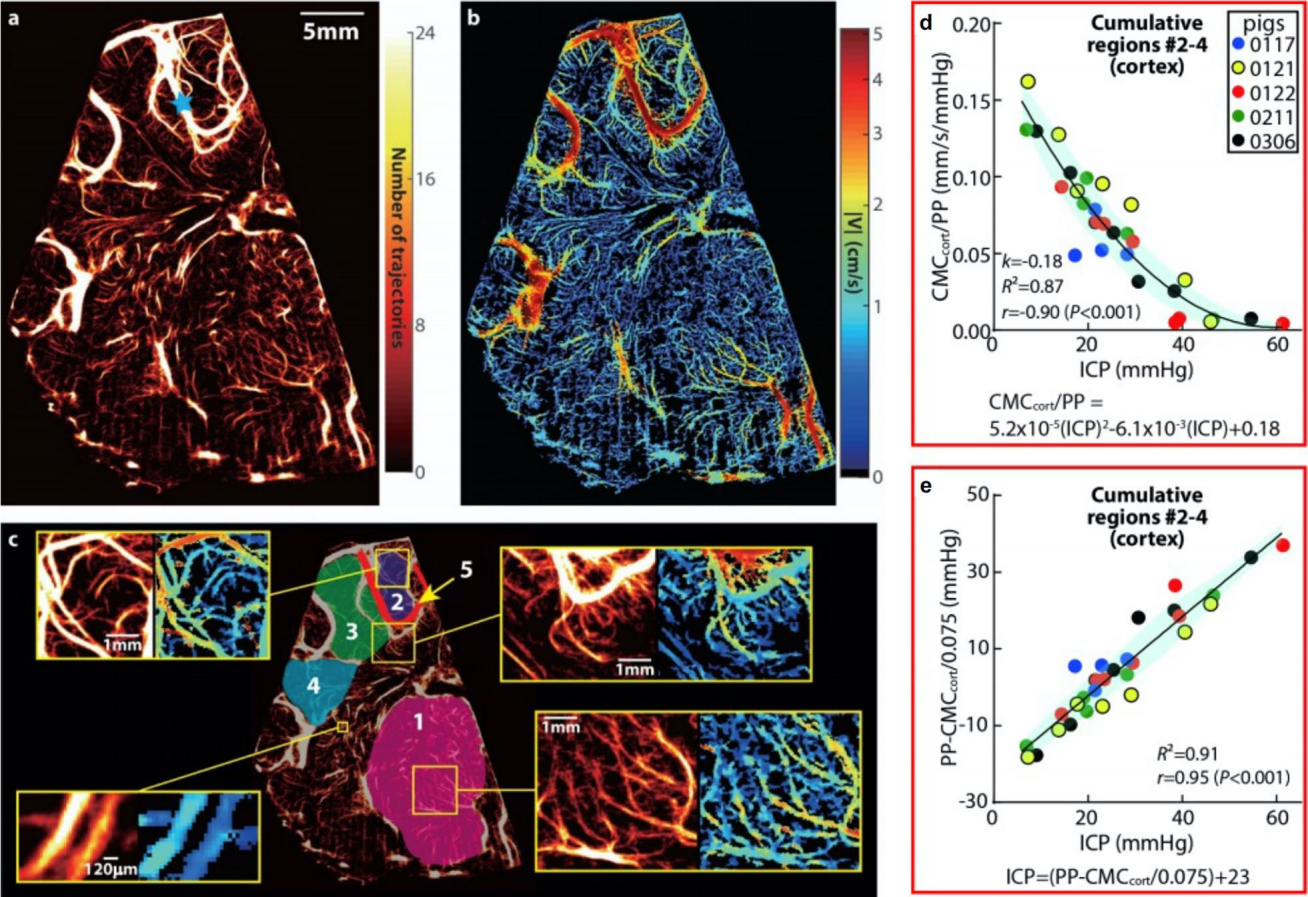
Cerebral vascular map and velocity distribution for a porcine hydrocephalus model at the baseline ICP. **a** A heatmap of all the trajectories containing at least 4 exposures visualizing the micro- and macro-vascular distributions in a coronal plane. The blue star marks the location of pulsed-wave Doppler ultrasound measurement. **b** The corresponding time-averaged velocity distribution. **c** Several sub-regions are labelled for statistical analysis of perfusion, including the micro-vessels in the thalamus (#1), several parts of the cortex (#2, #3, and #4), as well as a macro blood vessel (#5). A few regions are magnified to provide a closer view of the current regions of interest. Sample velocity profiles across two different blood vessels are also provided in **c**. **d**, **e** Relationships between the ICP and the Cerebral Microcirculation (CMC) parameter of the cortical regions #2–4. The CMC parameter is calculated by summing the time-averaged velocity (in mm/s) in each pixel of the area occupied by micro-vessels, and dividing the result by the total area of these regions. The pulse pressure (PP) is systolic blood pressure minus diastolic blood pressure

Brain ultrasound is used to image infants with hydrocephalus via the fontanelles. Ventriculodilitation can decrease cerebral blood flow, leading to ischemia. Intracranial pressure probes are the gold standard for monitoring, but they carry the risk of hemorrhage and infection, limiting their use. Dr. Misun Hwang of Children’s Hospital of Philadelphia discussed the use of CEUS in neonatal hydrocephalus. Transcranial Doppler can assess microvascular flow, and CEUS complements evaluation by allowing visualization of the whole brain and evaluation of microvascular flow. In a pig model, a burr hole was made in the skull to mimic a fontanelle, then artificial CSF injection into the ventricles was used to increase ICP from 0 to 60 mmHg (Fig. 6). Thus, with more research, better fetal hydrocephalus modeling may be on the horizon.

Dr. Kassa Darge of Children’s Hospital of Philadelphia discussed a global perspective on CEUS imaging in pediatrics. Publications on pediatric contrast ultrasound have been increasing, including the first book on contrast enhanced ultrasound in pediatric imaging. Pediatric CEUS is expanding in clinical use, research, and publication. Dr. Darge also discussed the first pediatric contrast ultrasound compendium, developed to discuss on-label CEUS uses, off label applications, and review CEUS uses in adults to suggest possible future applications in pediatrics.

In summary, the 35th Annual Advances in Contrast Ultrasound Meeting continues to provide an excellent resource for clinicians, engineers, basic scientists, sonographers, nurses, government officials, attorneys, and industry and patient partners on new advances in CEUS and its applications. It continues to demonstrate CEUS as a safe and effective diagnostic tool for imaging throughout the body. CEUS is a radiation free and cost-effective option for many indications. There is new research and progress for expanding CEUS use for cardiac imaging. Research into sonothrombolysis, for STEMIs and other clots, has had significant progress since the last conference. There has been progress in solid tumor imaging in the liver and kidneys, as well as surveillance of liver masses after ablation. Lymphatic imaging and sentinel lymph node identification is an exciting area of ongoing research. Finally, there has been much progress in pediatric brain imaging, and there is now a pediatric contrast ultrasound compendium. Come join this year’s meeting, which will be on September 1 and 2 in Chicago, IL, USA.

## Data Availability

Not applicable.
